# The effectiveness of dry needling in patients with chronic low back pain: a prospective, randomized, single-blinded study

**DOI:** 10.1038/s41598-022-19980-1

**Published:** 2022-09-22

**Authors:** Joanna Rajfur, Katarzyna Rajfur, Łukasz Kosowski, Karolina Walewicz, Robert Dymarek, Kuba Ptaszkowski, Jakub Taradaj

**Affiliations:** 1 Institute of Health Sciences, University of Opole, 45-060 Opole, Poland; 2 Faculty of Medical Sciences, Medical University of Silesia, 40-055 Katowice, Poland; 3 Department of Physiotherapy, Faculty of Health Sciences, Wroclaw Medical University, 50-355 Wrocław, Poland; 4 Institute of Physiotherapy and Health Sciences, Academy of Physical Education, 40-065 Katowice, Poland

**Keywords:** Pain management, Placebo effect, Rehabilitation, Randomized controlled trials

## Abstract

Dry needling (DN) is a standard procedure for treating musculoskeletal disorders. However, there are no clear recommendations for using DN in low back pain (LBP). Therefore, this study aimed to assess the effectiveness of the novel DN program for reducing pain intensity and improving functional efficiency in patients with chronic LBP. A group of 40 patients with chronic LBP due to the L5-S1 discopathy were eligible and randomized into experimental (n = 20) and control (n = 20) groups. The DN program was performed for the experimental group according to the Five Regulatory Systems (FRS) concept. The control group received sham therapy using placebo needles. DN sessions were performed twice a week for 4 weeks. A single needling application lasted 60 min. Both groups received standard treatment and physical exercise of LBP for 1 month. Subjective pain was measured by a visual analog scale (VAS), functional efficiency was assessed with the Oswestry Disability Index (ODI), and the lower spine range of motion was measured with the Schober test. There were significant differences in pain reduction (VAS) in both groups (*p* < 0.001). The strongest analgesic effect in the DN group yielded 6.45 points immediately after the therapy, 6.2 points after 1 month, and 6 points after 3 months. The DN group scored higher VAS reduction than the control group (*p* < 0.001). There were significant differences in the functional state (ODI) in the experimental group (*p* < 0.001). There was a significant ODI decrease by 18.1 points, after 1 month by 18.9 points, and after 3 months by 17.6 points. No significant differences were found in the control group (*p* > 0.05). Intergroup differences were observed in the functional efficiency in ODI in all measurement time-points (*p* < 0.001). There were significant differences in the range of motion (Schober test) in the DN group (main effect: *p* < 0.001). For all measurements, differences (*p* < 0.001) were observed in favor of DN compared to the control. In conclusion, DN program according to the FRS concept stands for the novel treatment method supplemented by an exercise program, effectively reducing pain and improving functional efficiency in LBP patients.

## Introduction

Dry needling (DN) is a standard procedure in treating musculoskeletal disorders. It penetrates the skin, subcutaneous tissues, and muscles using a thin needle. This puncture technique causes local muscle tremors, which relaxes the treated area. Hence, the DN treatment can reduce local tension and pain^1–3^. However, following the Evidence-Based Medicine approach, the effectiveness of DN in everyday practice seems to be insufficient and not well recognized, as shown by two reliable systematic reviews with meta-analyses.

In 2018, Hu et al.^4^ analyzed 16 randomized controlled trials (RCTs) on treating chronic low back pain (LBP) with DN. The analgesic effects and improved functional efficiency were confirmed, although these findings seemed to be weak because of the low methodological quality of some reports and the variety of application techniques and treatment durations. The authors included mainly studies with the myofascial trigger point (MTrP) DN techniques. The following systematic review and meta-analysis identified the trigger point using palpation. A pincer grip technique was employed to lift the skin gently. Additionally, flat palpation was utilized to take up the slack of the skin. A high-quality, sterile, disposable, solid filament needle was inserted directly through the skin or using a guide tube that is then removed. The depth of needle penetration was sufficient to engage the MTrP. Once the needle has penetrated the skin and is inserted into the muscle, techniques vary: the practitioner utilized a slow, steady, lancing or postoning motion in and out of the muscle (termed dynamic needling) or left the needle in situ (termed static needling), or the needle was rotated several revolutions in order to draw the fascia or soft tissues. There is no consensus as to which technique is ideal.

Liu et al.^5^ also published in 2018 a systematic review with a meta-analysis on DN of MTrP in patients with LBP. This work included 11 RCTs, and for a combined population of 802 patients, “moderate” evidence for the effectiveness of both static and dynamic DN trigger points in short-term pain relief was documented. However, the meta-analysis showed that findings on DN are inconclusive regarding patients’ physical fitness, quality of life, and long-term treatment effects.

There are no clear recommendations for the DN application technique, suggesting the rationale for prospective RCTs with placebo effect estimation, multiple measures, and follow-up. The innovative and popular method—often used by practitioners—is the Five Regulatory Systems concept (FRSc) idea. Unfortunately, commercial use is ahead of reliable research studies. The basic science underlying this idea is relatively poor. There are no well RCTs as well. According to practitioners, the method draws attention to the tissues of the fascia, which can structurally adapt to external forces^6^. The punctured tissue undergoes relaxation, resulting from a response to numerous nerve endings, relaxing the point under pressure, along with the adjacent area. This process forms the basis of the first regulatory system. The second regulatory system is associated with stasis, or impaired flow of blood, lymph, and extracellular fluid in each compartment, with its etiopathogenesis resembling that of compartment syndrome^7^.

Applications targeting drainage and fluid return from the affected area may result in pain reduction. The third regulatory system refers to the influence of the autonomic nervous system on the organ of locomotion and the antagonism of the action of its two main branches, i.e., the sympathetic and parasympathetic branches. According to the FRSc principles, an increased sympathetic impulse can lead to significant or even complete reversal of pain, often combined with increased range of motion and return of previously impaired function. The fourth regulatory system involves proprioception and the extrapyramidal control of voluntary movements. The focus of therapy in the FRSc concept is on restoring proper proprioceptive stimulation and, through targeted action, restoring proper muscle balance. Due to the deep sensory receptors responsible for proprioception, it is possible to orient the body in space. Impaired local or systemic proprioception in the musculoskeletal system can affect movement pattern impairments^8,9^. The fifth regulatory system suggests the presence of reactive skin zones over the affected areas.

Therefore, the present study aimed to assess the effectiveness of the novel DN program according to the FRSc for reducing pain intensity and improving functional efficiency in patients with symptoms of chronic LBP.

## Results

**Figure 1:**
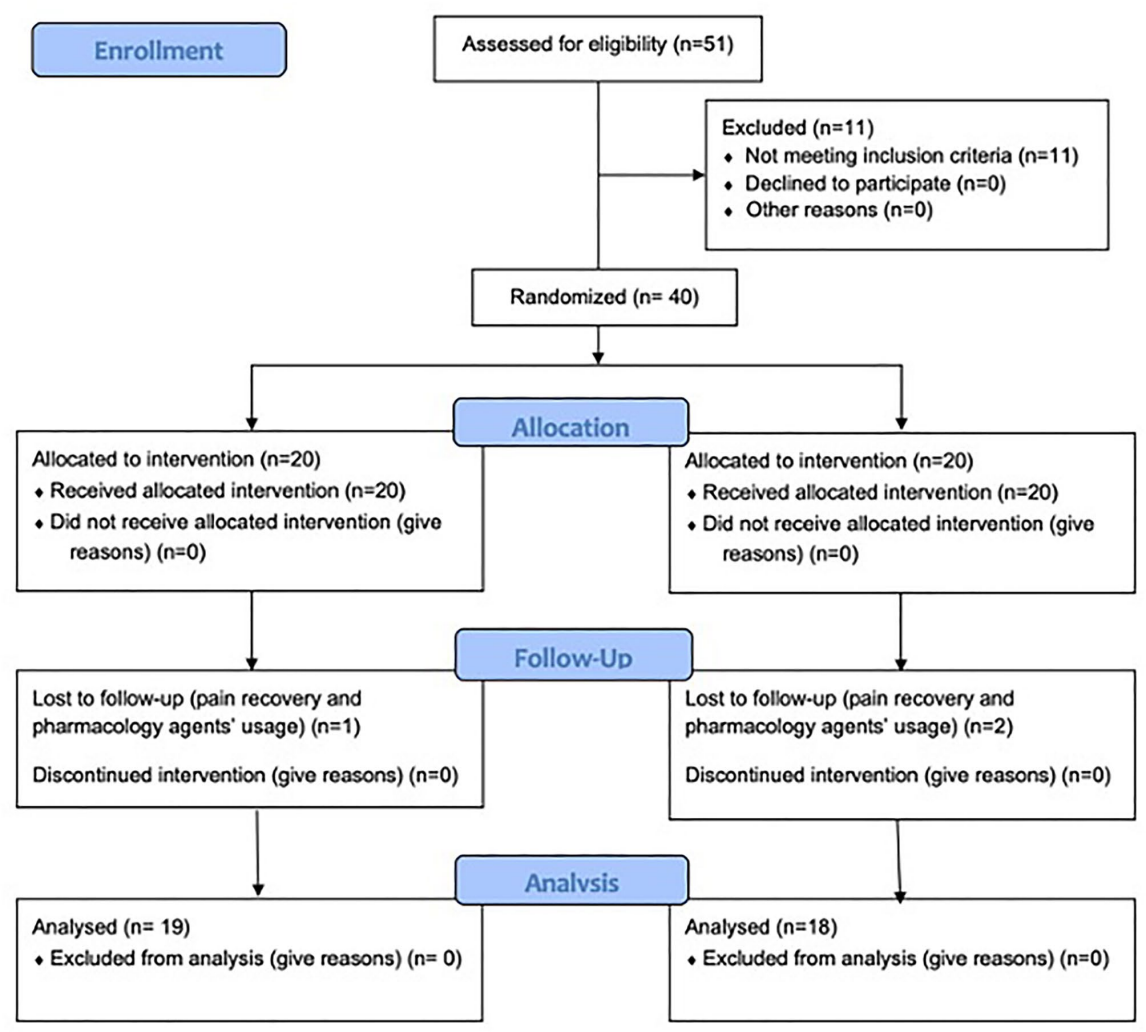
CONSORT flow chart of study participants.

Table 1 shows the demographic characteristics of the patient sample. There were no significant differences between the experimental (n = 20) and control groups (n = 20), and the groups were homogeneous in terms of basic parameters. The flow of participants in the project stages is shown in Fig. 1, in line with the CONSORT 2010 guidelines.

**Table 1:** Characteristics of study participants.

Variable	Experimental group (n = 20)							Control group (n = 20)							*p*-value
	x̅	Me	Min	Max	Q1	Q3	SD	x̅	Me	Min	Max	Q1	Q3	SD	
Age [years]	52.7	54.0	39.0	76.0	45.0	62.0	17.7	56.8	59.5	36.0	74.0	47.0	64.0	11.5	0.095*
Body weight [kg]	72.5	72.5	57.0	88.0	61.5	83.0	10.9	72.3	71.5	54.0	92.0	62.5	82.5	11.7	0.957*
Body height [cm]	172.9	171.0	163.0	186.0	166.0	178.0	6.8	167.7	170.0	152.0	181.0	159.0	175.0	8.6	0.102*
BMI [kg/m2]	24.2	23.7	19.9	30.1	21.8	27.1	3.3	25.6	26.0	19.0	30.1	22.8	28.1	3.2	0.185*
Disease duration [months]	56.5	47.0	3.0	199.0	23.0	85.0	49.9	59.2	49.0	3.0	174.0	23.6	78.0	50.3	0.967*
Sex [F/M]	F—n = 9; 45.0% M—n = 11; 55.0%							F—n = 12; 60.0% M—n = 8; 40.0%							0.342**

*n* number of individuals, x̅, mean; *Me* median, *Min* minimum value, *Max* maximum value, Q1, lower quartile; Q3, upper quartile, *SD* standard deviation, *F* female, *M* male.

*Mann-Whitney U test; **chi-square test.

The results were analyzed for both groups in consecutive measurements: before the therapy (M1), after the therapy (M2), 1 month (M3) and 3 months after the end of the therapy (M4). Table 2 and supp. Table 1 show comparisons of perceived pain (VAS scale) in the DN and control groups. There were significant differences in both groups (*p* < 0.001). The strongest analgesic effect in the DN group yielded 6.45 points immediately after the therapy, 6.2 points after 1 month, and 6 points after 3 months. A significant analgesic effect of 2.25 points was obtained immediately after the therapy in the control group. Long-term measurements indicated a gradual deterioration of perceived pain (on average by 2.35 points after 1 month and 2.3 points after 3 months).

**Table 2:** The VAS scores in both groups (points).

Variable	Measure	Experimental group (n = 20)							Control group (n = 20)						
		x̅	Me	Min	Max	Q1	Q3	SD	x̅	Me	Min	Max	Q1	Q3	SD
VAS [score]	Before	8.10	8.00	7.00	10.00	7.50	9.00	0.9	8.15	8.00	7.00	10.00	7.00	9.00	1.0
	After	1.65	1.50	1.00	5.00	1.00	2.00	0.9	5.90	6.00	4.00	7.00	5.00	7.00	1.1
	1 month FU	1.90	1.50	1.00	6.00	1.00	2.00	1.4	8.25	8.00	7.00	10.00	7.50	9.00	1.0
	3 months FU	2.10	1.50	1.00	7.00	1.00	3.00	1.6	8.20	8.00	6.00	10.00	7.50	9.00	1.1
*p*-value (main effect*)		** < 0.001**							** < 0.001**						
*p*-value (main effect**)		Before versus after: ***p* < 0.001** Before versus 1 month: ***p* < 0.001** Before versus 3 months: ***p* = 0.001** After versus 1 month: *p* = 0.135 After versus 3 months: ***p* = 0.025** 1 month versus 3 months: ***p* = 0.042**							Before versus after: ***p* < 0.001** Before versus 1 month: *p* = 0.163 Before versus 3 months: *p* = 0.577 After versus 1 month: ***p* < 0.001** After versus 3 months: ***p* < 0.001** 1 month versus 3 months: *p* = 0.330						

Significant values are in [bold].

*n* number of individuals, x̅, mean, *Me* median, *Min* minimum value, *Max* maximum value, Q1, lower quartile, Q3, upper quartile; *SD* standard deviation, *FU* follow-up.

*Friedman's ANOVA; **Dunn’s test.

**Figure 2:**
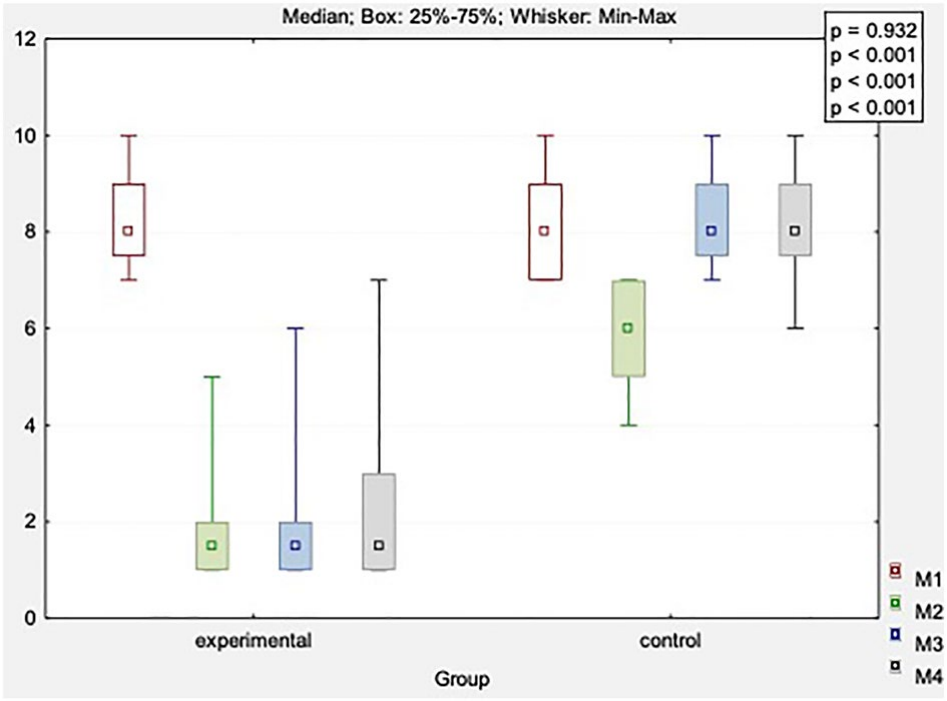
Comparison of changes in pain scores (VAS) obtained in four measurements between study and control group. M1, before treatment; M2, after treatment; M3, 1 month after study; M4, 3 months after study.

Between-group comparisons of pain (experimental vs. control groups) were significant for all time intervals (immediately after treatment, one and 3 months after treatment; see Fig. 2, main effect *p* < 0.001). In addition, the DN group scored higher on the VAS scale than the control group (all *p* values < 0.001), indicating the clinical advantage of DN over sham procedures and the occurrence of an analgesic effect immediately after treatment and in long-term follow-up conditions.

Table 3 and supp. Table 2 present comparisons of the ODI in both study groups. There were significant differences in the test group (*p* < 0.001). Positive changes were found after the end of treatment, as indicated by a significant decrease by 18.1 points, after 1 month by 18.9 points, and after 3 months by 17.6 points. This suggested that the DN technique contributed to the functional improvement of patients with LBP. No significant differences were found in the control group (*p* > 0.05).

**Table 3:** The ODI scores in both groups.

Variable	Measure	Experimental group (n = 20)							Control group (n = 20)						
		x̅	Me	Min	Max	Q1	Q3	SD	x̅	Me	Min	Max	Q1	Q3	SD
ODI [score]	Before	36.0	35.5	29.0	47.0	33.0	38.0	4.5	39.0	41.0	29.0	50.0	33.0	43.5	7.3
	After	17.9	14.5	9.0	31.0	12.0	23.5	7.4	36.1	34.0	28.0	50.0	29.5	41.5	6.7
	1 month FU	17.1	14.0	9.0	31.0	12.0	23.0	7.0	38.4	39.0	29.0	50.0	31.0	43.5	7.4
	3 months FU	18.4	16.0	11.0	31.0	12.0	25.0	6.9	39.0	39.5	29.0	50.0	33.0	43.5	6.9
*p*-value (main effect*)		** < 0.001**							0.242						
*p*-value (multiple comparisons**)		Before versus after: ***p* < 0.001** Before versus 1 month: ***p* < 0.001** Before versus 3 months: ***p* < 0.001** After versus 1 month *p* = 0.251 After versus 3 months: *p* = 0.569 1 month versus 3 months: *p* = 0.053							**–**						

Significant values are in [bold].

*n* number of individuals, x̅, mean, *Me* median, *Min* minimum value, *Max* maximum value, Q1, lower quartile; Q3, upper quartile; *SD* standard deviation, *FU* follow-up.

*Friedman’s ANOVA; **Dunn’s test.

**Figure 3:**
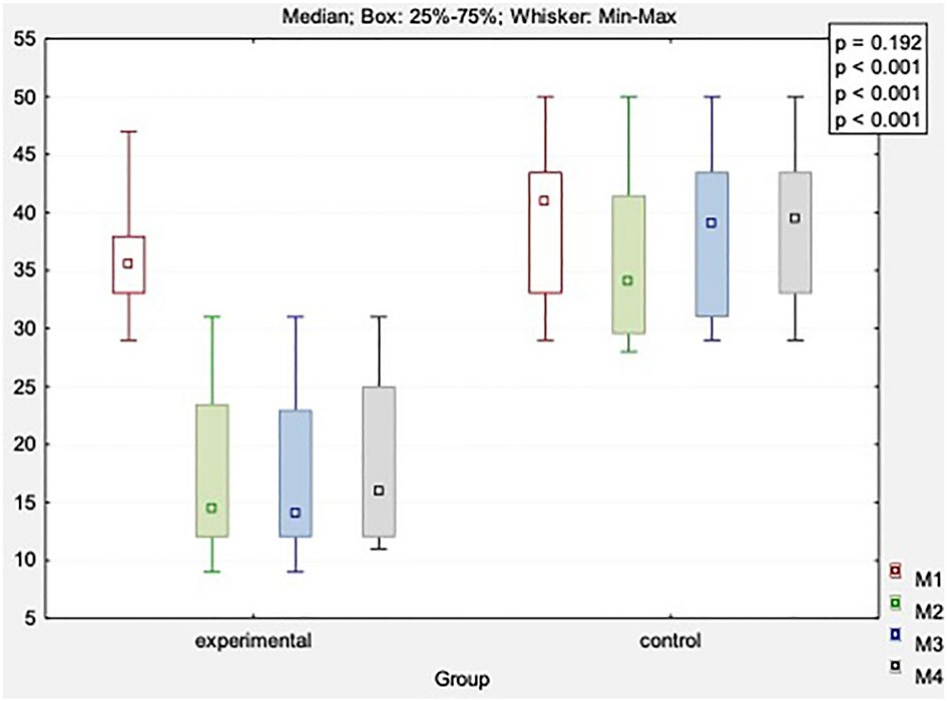
Comparison of changes in functional status (ODI) obtained in four measurements between study and control group. M1, before treatment; M2, after treatment; M3, 1 month after study; M4, 3 months after study.

There were group differences in the functional efficiency measured by the ODI scale (main effect *p* < 0.001) after the completion of the treatment, 1 month, and 3 months after the treatment, respectively. In addition, there were improvements in the functional status and clinical effects after applying the DN procedure to the control group (Fig. 3).

Table 4 and supp. Table 3 show the Schober test results for the experimental and control groups. There were significant differences in the DN group (main effect: *p* < 0.001). In addition, there was an increase in the test group immediately after the treatment by 1.8 points, 1 month after the treatment by 1.7 points, and 3 months after the treatment by 1.5 points. No significant differences were found in the control group (*p* > 0.05).

**Table 4:** The Schober’s test results in both groups.

Variable	Measure	Experimental group (n = 20)							Control group (n = 20)						
		x̅	Me	Min	Max	Q1	Q3	SD	x̅	Me	Min	Max	Q1	Q3	SD
Schober’s test [cm]	Before	3.00	3.00	2.00	4.00	3.00	3.00	0.6	2.80	3.00	2.00	4.00	2.25	3.00	0.7
	After	4.80	5.00	4.00	6.00	4.00	5.00	0.6	2.65	2.50	2.00	4.00	2.00	3.00	0.6
	1 month FU	4.70	5.00	4.00	6.00	4.00	5.00	0.7	2.88	3.00	2.00	4.00	2.50	3.00	0.6
	3 months FU	4.50	4.00	3.00	6.00	4.00	5.00	0.8	2.65	2.50	2.00	4.00	2.00	3.00	0.7
*p*-value (main effect*)		** < 0.001**							0.188						
*p*-value (multiple comparisons**)		Before versus after: ***p* < 0.001** Before versus 1 month: ***p* < 0.001** Before versus 3 months: ***p* < 0.001** After versus 1 month: *p* = 0.163 After versus 3 months: ***p* = 0.010** 1 month versus 3 months: ***p* = 0.042**							–						

Significant values are in [bold].

*n* number of individuals, x̅, mean, *Me* median, *Min* minimum value, *Max* maximum value, Q1, lower quartile, Q3, upper quartile, *SD* standard deviation, *FU* follow-up.

*Friedman’s ANOVA; **Dunn’s test.

**Figure 4:**
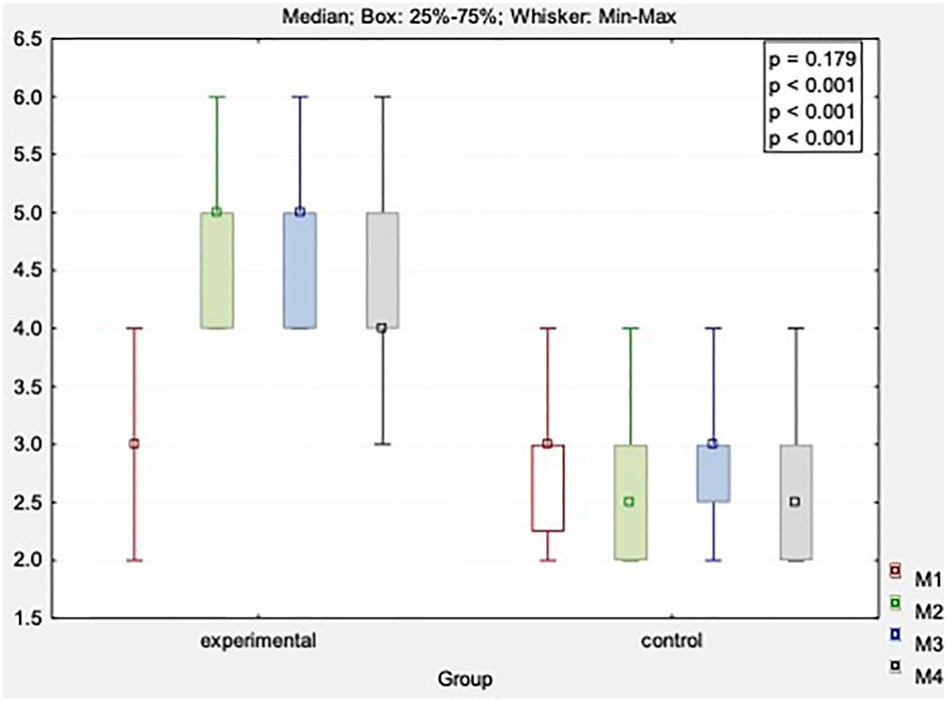
Comparison of changes in Schober’s test obtained in four measurements between study and control group. M1, before treatment; M2, after treatment; M3, 1 month after study; M4, 3 months after study.

Then, the four Schober test results were compared between the test and placebo groups (Fig. 4). For all measurements (immediately after treatment, one and 3 months after its completion), there were differences (main effect *p* < 0.001) observed in favor of DN compared to the sham condition. This suggested positive outcomes for the spine and its increased mobility in the lumbar region (both in short-term observations and long-term follow-up).

To sum up, the management of the DN technique in the test group according to FRSc recommendations led to substantial improvements in pain perception and functional status of patients. Furthermore, the positive clinical outcomes were observed immediately after the completion of the therapy and confirmed by the long-term follow-up measures, i.e., 1 and 3 months after the treatment.

In the control group, pain perception decreased only immediately after the therapy (short-term effect), while the long-term measures (1 and 3 months) indicated its gradual deterioration. No significant improvement was shown in the functional status, even after standard motor rehabilitation.

## Discussion

There are recent reports on DN for pain in the lower spine, but the credibility of some studies seems low. In addition, reports show that positive outcomes are usually validated by a single DN application, limiting this technique in everyday clinical practice where a series of treatments should be used. Finally, it is worth noting that a single DN application in our study took 60 min, which could be a potential challenge to the feasibility of the study.

Some studies include no comparisons with sham procedures or provide no evidence of long-term effects of DN, which hinders its clinical usefulness. To the best of our knowledge, the present study is the first to assess the effectiveness of DN treatments according to the FRSc recommendations. We tentatively suggest other centers conduct their RCTs according to a present methodology and justify the current results. At this stage, one can only refer our results to the existing reports, where, however, a variety of procedures and single DN applications were tested.

For instance, Martín-Corrales et al.^10^ examined the DN effectiveness and measured the long-term effects of treatment in combination with a 4-week LBP exercise program. The participants were randomly assigned to the experimental DN (23 people) and sham-DN (23 patients) groups. Patients underwent only one DN or sham-DN session before starting the 4-week exercise program. In the present study, patients received a total of 8 treatments over 4 weeks (twice a week). The pain was assessed using the VAS scale and a disability measure by the Roland-Morris Questionnaire (RMQ) scale. Measurements were taken before and after DN, after the exercise program, and 3 months after therapy. At the end of treatment and after a 3-month follow-up period, pain reduction was demonstrated for both groups, suggesting pain reduction in the DN condition. A similar methodology was used in the present study. On the contrary, the Spanish study showed changes in RMQ disability measures in both the DN and sham groups; however, no between-group differences were found. Our study showed that the DN group had better physical fitness and range of motion results.

Interestingly, the high effectiveness of the single DN application was shown by Wang-Price et al.^11^. It was demonstrated that a single DN session could reduce the sensitivity to pressure pain in LBP patients immediately after surgery and 1 week later. The study was conducted in the manipulated DN and non-manipulated DN groups in the lumbosacral segment of the spine (groups included 21 people each). Participants received one session assigned to the DN intervention. The Numeric Pain Rating Scale (NPRS) results were measured by the 11-point Likert scale, where 0 indicated 'no pain' and 10 was the worst pain imaginable, electromyography (EMG) amplitude, and pressure pain threshold (PPT). The study showed significant pain reduction immediately after DN treatment (*p* = 0.001) and 1 week after (*p* = 0.019); (a more substantial decrease in the NPRS measures was found immediately after treatment than after 1-week assessment). Our study employed different measurements that indicated improvements in the DN group for all parameters after treatment, 1 and 3 months after its completion (VAS, ODI, ROM: *p* < 0.001). However, the study in the US setting provides no long-term results, undermining the effectiveness of a one-time DN application for permanent remission of the disease.

A slightly larger number of DN applications are presented by researchers in clinical trials on trigger point stimulation. Thus, this is another treatment option, although the meta-analysis by Chinese researchers (5) reports many limitations.

Téllez-García et al.^12^ examined DN treatment of LBP patients assigned to two conditions: the trigger point DN treatment (TrP-DN, n = 6) and the TrP-DN combined with neurological education (TrP-DN + EDU, n = 6). Participants received 3 TrP-DN sessions once a week. Patients from the TrP-DN + EDU group also received two 30-min educational sessions once a week for the last 2 weeks of therapy. The study used several questionnaires (the NPRS, RMS, ODI, Tampa Scale of Kinesiophobia—TSK) and measured the pressure pain threshold (PPT). 1 week after therapy, control measurements were taken. For both groups, the TrP-DN technique was effective in short-term pain relief and short-term reduction of disability, kinesophobia, and pressure sensitivity. However, the study did not investigate the long-term effects of DN.

Tüzün et al.^13^ studied a sample of 34 patients with LBP by assigning them to the DN group with classic massage (DN + M) and the second group that included hot compresses, TENS, ultrasounds, and exercise program (CPT). There were six treatment sessions (twice a week for 3 weeks). The primary pain measurement was performed with the McGill Pain Questionnaire and the VAS scale, while the secondary measures were taken with the Beck Depression Inventory (BDI) and TSK scales. Measures were analyzed before and after treatment, and significant differences were found in both groups (all *p* < 0.05). The above results confirmed the effectiveness of DN, although the effects were short-term and the treatment was heterogeneous across the groups.

### Clinical implications

The present study proposes a new DN method for the clinical application of LBP, ensuring effective pain management in the lower spine. Our observations show that DN therapy is more beneficial than the standard exercise program, complementing it. Therefore, the presented DN procedure can be addressed to practitioners interested in extending the primary treatment of DN.

### Study limitations

According to the literature, our RCTs are the first to use DN for LBP treatment following the FRSc approach. In line with the FRSc recommendations, DN planning should involve homogeneous research material, sample blanking, consistent qualification criteria, and scrutiny of short- and long-term results. This guarantees the clinical effectiveness of DN and furnishes the standard physiotherapy program with novel treatment opportunities. Our findings seem promising, but these results should be further verified to evaluate the clinical utility of treatment more fully in LBP patients. Despite some statistical trends, we found no improvements in motion ranges in the lumbosacral spine and no evidence of a reversal in disability after standard physiotherapy and sham-DN treatments. Presumably, performing the training by all participants under the supervision of the same therapist could enhance clinical outcomes, but for organizational reasons, this was not feasible in the study. Perhaps patient education might have also contributed to better performance in the study. In our study, all procedures (DN and sham) were applied by the same therapist; however, they were made in different parts of campus. The participants could not be informed about allocation, but they had contact with this therapist. Besides, participants were not asked at the end of the study to report their perception of which group they belonged to, which is also a potential methodological limitation. Although most studies instead recommend the individual approach to the patient. Other studies should also extend methodology by including objective measurements, assessing measures of correct body posture, biomechanical gait analysis, and increasing sample size and follow-up periods (6 and 12 months). We based only on the Patient-Reported Outcome Measures (PROMs). Other treatment protocols as primary rehabilitation in the control group may be considered in the future.

## Conclusions

DN is the novel treatment method supplemented by an exercise program, effectively managing LBP symptoms. Thus, the proposed treatment procedure in this study can reduce pain and improve functional efficiency in LBP patients.

## Methods

### Study design

The research project was carried out in the Clinical Research Laboratory of the Institute of Health Sciences at the University of Opole, Poland. The study was conducted in cooperation with the Department of Physiotherapy of the Wroclaw Medical University in Poland. The local Bioethics Committee of the University of Opole approved the research (no. KB/91/FI/2018). The study was registered in the International Standard Randomized Controlled Trial Number (ISRCTN) registry database (no. ISRCTN16627714). All participants gave written informed consent to take part in the study. The studies were conducted following the Helsinki Declaration and good clinical practice.

### Randomization and blinding

Patients were selected for the project by a research team consisting of an internist, radiologist, neurologist, neurosurgeon, orthopedist, and physiotherapist. The project was designed as a prospective, single-blinded RCT with follow-up observation. Participants were randomly allocated to comparative groups (experimental or control) by simple randomization with a 1:1 ratio through a computerized random sequence on the random.com website. The assignment to the group was independent of the time and the research staff performing the procedure. All tests and surveys were carried out by two physiotherapists, who trained the repeatability and accuracy of the techniques for 4 months before the start of the research. All DN treatments were performed by one experienced physiotherapist with an instructor license. Physical exercises were performed “one-on-one” and conducted by certified therapists who also had 4 months of training in advance to ensure uniformity of procedure. The research staff who carried out the therapy and measurements had no contact with the qualification team or those who analyzed the results. The same therapist applied both procedures (DN and sham); however, all procedures were made in different parts of campus. Therefore, the participants could not be informed about allocation.

### Sample size

The sample size of the present study was based on group differences in primary outcomes (means and standard deviations of pain experience), which were estimated at 20 participants (in each group). A 20% decrease in the sample size to follow-up was allowed for calculations. The sample size was estimated based on 10 randomly selected results at the design stage of the study (5 from each group). Means and standard deviations of the results of VAS (difference between results before and after intervention) were used to estimate the sample size (the estimated sample size for a two-sample t-test). Parameters of the experimental group: mean = 6.1 points, standard deviation SD = 1.75 points; parameters of the control group: mean = 4.4 points, standard deviation SD = 1.68 points. The alpha level was set at 0.05, and the power of the test at 0.8. It also assumed no correlation of evaluated variables and adopted a 2-sided null hypothesis. Sample size estimation was performed using Statistica 13 (TIBCO Software Inc., Palo Alto, USA).

### Participants

Patients with L5-S1 discopathy, and chronic pain (more than 3 months), who did not undergo spinal surgical intervention, were eligible for the project. The participants were of legal age and had valid MRI examinations confirming the diagnosis of the LBP syndrome (changes at the level of the III degree according to the Modic classification in the L5-S1 section). The qualified participants did not have any previous DN procedures (in general, applications in other sites).

The exclusion criteria were: no pain and reduced mobility in the lumbosacral section, other spinal conditions (spondylolisthesis, fractures, tumors, infection, rheumatic diseases, cauda equina syndrome), sciatica and radicular symptoms, pregnancy, implanted cardiac pacemaker, blood coagulation disorders, anticoagulant therapy, steroid therapy, metal implants within the application site, sensory disturbances, mental disorders, cancer, skin changes within the treatment site, viral and bacterial infections, fever, exhaustion, untreated hypertension, taking painkillers and anti-inflammatory drugs, fear of needles, no consent to the procedure.

### Treatment

**Figure 5:**
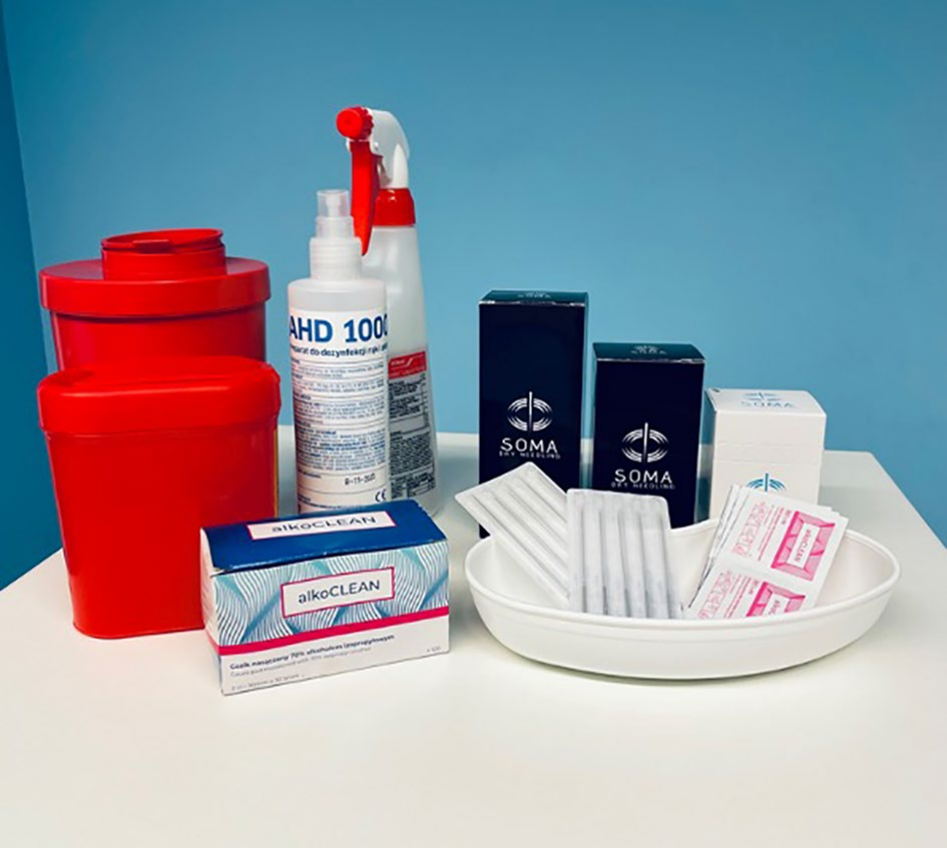
Equipment for DN procedure.

**Figure 6:**
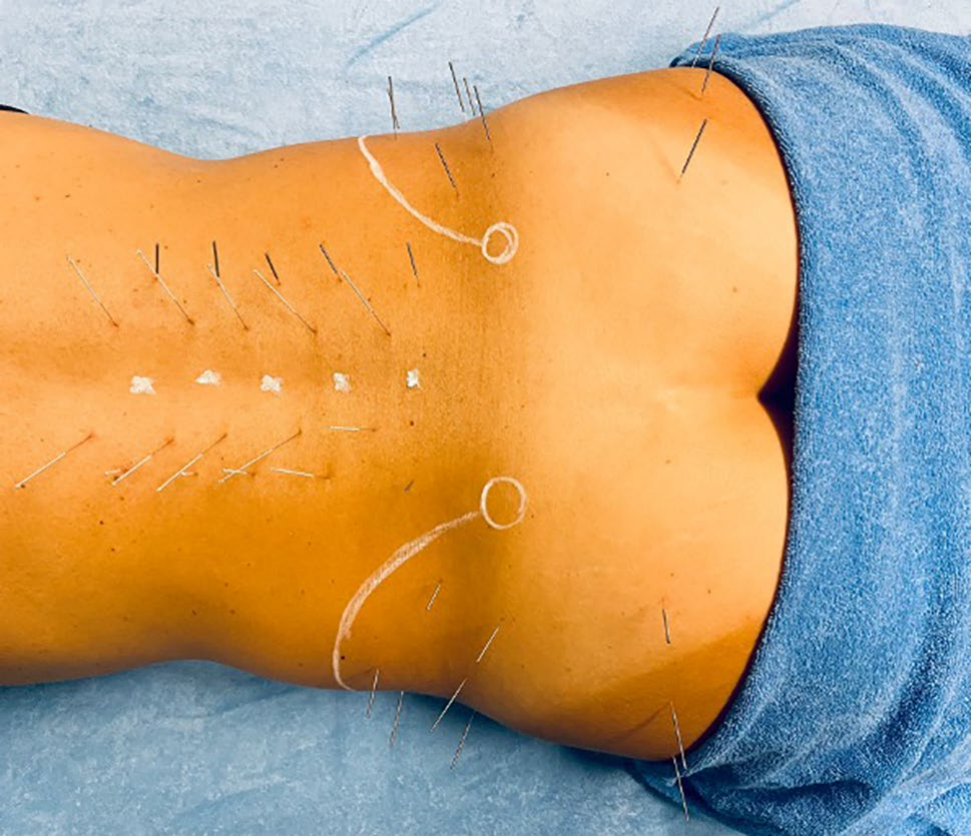
DN application in the experimental group. In the figure, as topographic reference points, the sites of the spinous processes of the vertebrae, the outlines of the iliac crests, and the posterior superior iliac spines are sketched in white.

For the experimental group, the study sued sterile, disposable SOMA needles made of Japanese surgical steel with a thickness of 0.3 mm and a length adapted to the punctured area (Fig. 5). The entire DN program is shown in Fig. 6. A single treatment included the following activities:

- Needling is activated in the furrow area between the spinous processes of the spine and the course of the dorsal extensor muscle. It was applied to palpable thickenings within the soft tissue on the L1–L5 spine segment on both sides (the puncture site slightly above and laterally from the given structure). The direction of the needle was spinal and caudal, with an inclination of 30° from the surface of the skin until the needle rested against the body of the vertebra below. The technique of work was “pumping” (rhythmic extension and insertion of the needle against the skin surface without removing it altogether) to stimulate (induce a pain response). After a few strokes of the needle, there was a pause of approximately 15 min (during which the application was not interfered with) and needle movements were repeated until the end of the procedure or when the pain response to “pumping” disappeared. The length of the needles was 75 mm.
- Needling is applied within palpable connective tissue bands arranged transversely on the mass of the dorsal extensor muscle. The structures felt on the L1-L5 section, on both sides, were needled. The direction of the needle was ventral (perpendicular to the skin surface) until a characteristic resistance was felt (approx. 1.5–2 cm deep). The work technique was to twist the needle to one side until it stopped. Then there was an interruption of approximately 15 min (during which the application was not interfered with), and the needle movements were repeated until the treatment time was finished or the needle stopping effect was unnoticed when twisting. The needle's length was equal to 30 mm.
- Needling activated in the neurocompartment of the superior gluteal nerve through punctures and local twitch response (LTR) generation within the palpable sensitive sites of possible junctions of the superior gluteal nerve (both upper and lower branches) with the muscles: gluteus great, gluteus middle, and tensioner of the broad fascia. The direction of the needle movement was ventral (perpendicular to the skin surface), with a characteristic densification feeling (giving greater resistance under the needle). The application technique was "pumping" to a minimum of two contractions, followed by an interruption of approximately 15 min (during which the application was not interfered with) and needle movements were repeated until the end of the treatment time or the disappearance of the systolic response to “pumping”. The needle's length was equal to 100 mm.
- Needling activated in the myogelosis area of the pear-shaped muscle, palpable as a tender thickening of the muscle fibers jumping under the fingers. The direction of the needle movement was ventral (perpendicular to the skin surface), with the feeling of resistance in the pear-shaped muscle. The technique involved “pumping” to at least two contractions, followed by an interruption of approximately 15 min (during which the application was not interfered with), and needle movements were repeated until the end of the treatment time or the disappearance of the systolic response to "pumping". The needle's length was equal to 100 mm.

**Figure 7:**
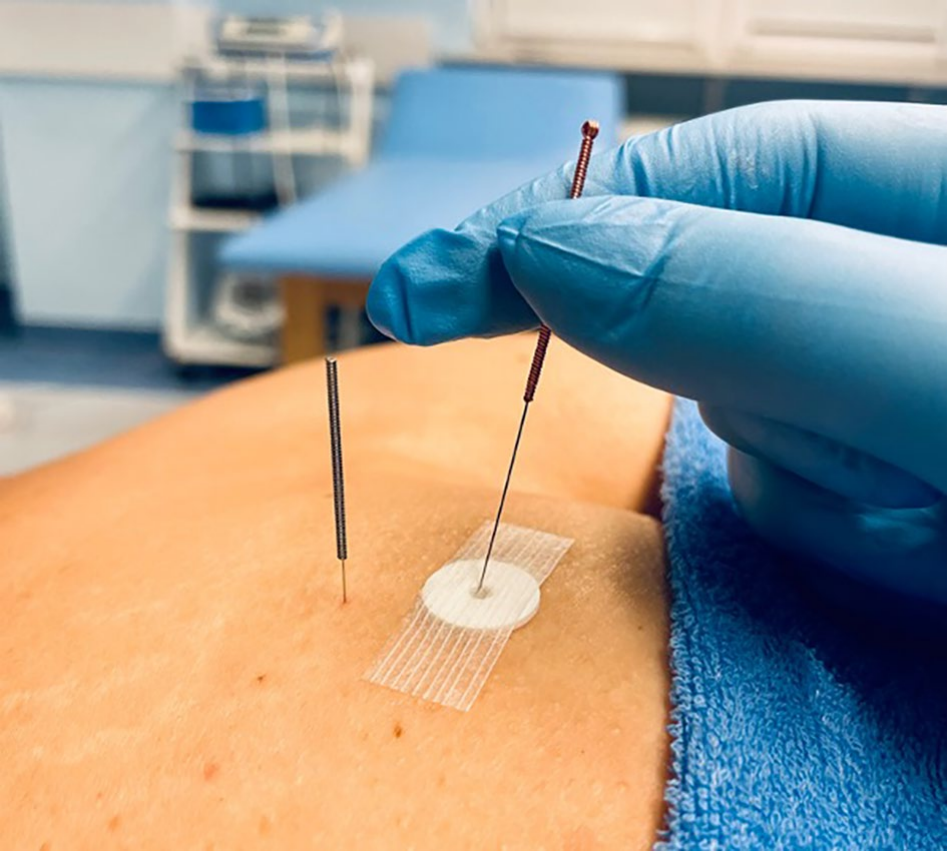
DN application in the control group using telescopic needle (sham therapy). For comparison, an actual needle inserted is presented next to the application site.

The control group received sham therapy using specialized placebo needles, with the telescopic design to be placed on the skin without piercing (see Fig. 7). Exact locations of placebo needles were used in the control and test groups, preceded by precise palpation. At designated points, unique rings were placed to ensure a distance from the skin. Next, the discs were fixed with thin slices, inserting telescopic placebo needles through them. This way, the needles stayed on the surface of the skin without penetrating it, allowing soft movements to mimic the therapy itself.

Treatments in groups were performed twice a week (Monday and Thursday) for 4 weeks (8 treatments in total). A single DN application lasted 60 min. The patients were informed about preparation for the procedure (i.e., ensuring clean skin, free of ointments and creams). The same aseptic measures were used in all groups. The treatments met the highest standards of disinfection and safety. The groups also received standard treatments for LBP and physical exercise for a month (45 min a day, five times a week, Monday through Friday). The stabilization training included myofascial relaxation of the spine extensors, activation of the lumbosacral complex and deep muscles, stimulation of proper breathing and proper activation of the transverse abdominal muscle, and postural and dynamic training. The procedures were provided due to recent clinical guidelines^14–16^.

### Measurements

Subjective pain sensations were measured by a visual analog scale (VAS), on which the patient indicated their pain intensity from levels 0 to 10 (0 indicates ‘no pain,’ 10 indicates ‘severest pain’ of actual value) in the LBP region^17^. Functional efficiency was assessed with the Oswestry Disability Index (ODI)^18^. The motion range of the lower spine was measured with the Schober test^19^. In both groups, measures were taken before and immediately after treatment. Long-term effects were measured 1 and 3 months after the end of therapy^20^. The participants did not use physiotherapy or pharmacotherapy treatments during the study period.

### Statistical analysis

Statistical analysis was performed using the Statistica 13 program (TIBCO, Inc., Palo Alto, USA). For measurable variables, means, medians, standard deviations, quartiles, and the range of variability with extreme values were calculated. Qualitative variables were expressed in frequencies (percentages). Quantitative variables were analyzed with the Shapiro–Wilk test to examine the normality of distribution. The between-group comparisons were made with the chi-square test (χ^2^). Within-group comparisons for measurements 1–4 were performed by Friedman's analysis of variance and a post-hoc test (Dunn's test). The between-group comparisons (experimental vs. placebo-controlled groups) were assessed using the Kruskal–Wallis analysis of variance in a post-hoc test (U-Mann–Whitney test with Bonferroni correction). The significance level of α = 0.05 was adopted for all analyzes.

### Ethical considerations

The study protocol was approved by the Local Ethics Committee of the University of Opole in Poland (approval no. KB/90/FI/2018).

## Supplementary Information

Supplementary Information.

## Data Availability

The data generated during this study are available within the article. Datasets analyzed during the current study preparation are available from the corresponding author on reasonable request.
