# Supplementary material for: The effectiveness of dry needling in patients with chronic low back pain: a prospective, randomized, single-blinded study
Source: Sci Rep. 2022 Sep 22;12:15803. doi: 10.1038/s41598-022-19980-1 (PMC9500077; doi:10.1038/s41598-022-19980-1)
Supplement: Supplementary file 1 — Supplementary Information. [file 41598_2022_19980_MOESM1_ESM.pdf]

**Supplementary Table 1.** The VAS scores in both groups (repeated measures ANOVA).

| **Variable** | **Measure** | **Experimental group (n=20)** | | | | | | | **Control group (n=20)** | | | | | | | ***p** Group*** | ***p** Measure*** | ***p** Group x Measure*** | **Eta$^2$** | **Cohen f** |
|---|---|---|---|---|---|---|---|---|---|---|---|---|---|---|---|---|---|---|---|---|
| | | $\bar{x}$ | Me | Min | Max | Q1 | Q3 | SD | $\bar{x}$ | Me | Min | Max | Q1 | Q3 | SD | | | | | |
| VAS [score] | Before | 8.10 | 8.00 | 7.00 | 10.00 | 7.50 | 9.00 | 0.9 | 8.15 | 8.00 | 7.00 | 10.00 | 7.00 | 9.00 | 1.0 | <0.001 | <0.001 | <0.001 | 0.57 | 1.12 |
| | After | 1.65 | 1.50 | 1.00 | 5.00 | 1.00 | 2.00 | 0.9 | 5.90 | 6.00 | 4.00 | 7.00 | 5.00 | 7.00 | 1.1 | | | | | |
| | 1 month FU | 1.90 | 1.50 | 1.00 | 6.00 | 1.00 | 2.00 | 1.4 | 8.25 | 8.00 | 7.00 | 10.00 | 7.50 | 9.00 | 1.0 | | | | | |
| | 3 months FU | 2.10 | 1.50 | 1.00 | 7.00 | 1.00 | 3.00 | 1.6 | 8.20 | 8.00 | 6.00 | 10.00 | 7.50 | 9.00 | 1.1 | | | | | |

***Abbreviations:*** n – number of individuals; $\bar{x}$ – mean; Me – median; Min – minimum value; Max – maximum value; Q1 – lower quartile; Q3 – upper quartile; SD – standard deviation; FU – follow-up. ***Notes:*** *repeated measures ANOVA.

**Supplementary Table 2.** The ODI scores in both groups (repeated measures ANOVA).

| Variable | Measure | Experimental group (n=20) | | | | | | | Control group (n=20) | | | | | | | ***p**** ***Group*** | ***p**** ***Measure*** | ***p**** ***Group x Measure*** | **Eta²** | **Cohen f** |
|---|---|---|---|---|---|---|---|---|---|---|---|---|---|---|---|---|---|---|---|---|
| | | $\bar{x}$ | Me | Min | Max | Q1 | Q3 | SD | $\bar{x}$ | Me | Min | Max | Q1 | Q3 | SD | | | | | |
| ODI [score] | Before | 36.0 | 35.5 | 29.0 | 47.0 | 33.0 | 38.0 | 4.5 | 39.0 | 41.0 | 29.0 | 50.0 | 33.0 | 43.5 | 7.3 | <0.001 | <0.001 | <0.001 | 0.24 | 0.54 |
| | After | 17.9 | 14.5 | 9.0 | 31.0 | 12.0 | 23.5 | 7.4 | 36.1 | 34.0 | 28.0 | 50.0 | 29.5 | 41.5 | 6.7 | | | | | |
| | 1 month FU | 17.1 | 14.0 | 9.0 | 31.0 | 12.0 | 23.0 | 7.0 | 38.4 | 39.0 | 29.0 | 50.0 | 31.0 | 43.5 | 7.4 | | | | | |
| | 3 months FU | 18.4 | 16.0 | 11.0 | 31.0 | 12.0 | 25.0 | 6.9 | 39.0 | 39.5 | 29.0 | 50.0 | 33.0 | 43.5 | 6.9 | | | | | |

***Abbreviations:*** n – number of individuals; $\bar{x}$ – mean; Me – median; Min – minimum value; Max – maximum value; Q1 – lower quartile; Q3 – upper quartile; SD – standard deviation; FU – follow-up. ***Notes:*** *repeated measures ANOVA.

**Supplementary Table 3.** The Schober's test results in both groups (repeated measures ANOVA).

| **Variable** | **Measure** | **Experimental group (n=20)** | | | | | | | **Control group (n=20)** | | | | | | | ***p**** ***Group*** | ***p**** ***Measure*** | ***p**** ***Group x Measure*** | **Eta$^2$** | **Cohen f** |
|---|---|---|---|---|---|---|---|---|---|---|---|---|---|---|---|---|---|---|---|---|
| | | $\bar{x}$ | Me | Min | Max | Q1 | Q3 | SD | $\bar{x}$ | Me | Min | Max | Q1 | Q3 | SD | | | | | |
| Shober's test [cm] | Before | 3.00 | 3.00 | 2.00 | 4.00 | 3.00 | 3.00 | 0.6 | 2.80 | 3.00 | 2.00 | 4.00 | 2.25 | 3.00 | 0.7 | <0.001 | <0.001 | <0.001 | 0.26 | 0.58 |
| | After | 4.80 | 5.00 | 4.00 | 6.00 | 4.00 | 5.00 | 0.6 | 2.65 | 2.50 | 2.00 | 4.00 | 2.00 | 3.00 | 0.6 | | | | | |
| | 1 month FU | 4.70 | 5.00 | 4.00 | 6.00 | 4.00 | 5.00 | 0.7 | 2.88 | 3.00 | 2.00 | 4.00 | 2.50 | 3.00 | 0.6 | | | | | |
| | 3 months FU | 4.50 | 4.00 | 3.00 | 6.00 | 4.00 | 5.00 | 0.8 | 2.65 | 2.50 | 2.00 | 4.00 | 2.00 | 3.00 | 0.7 | | | | | |

***Abbreviations:*** n – number of individuals; $\bar{x}$ – mean; Me – median; Min – minimum value; Max – maximum value; Q1 – lower quartile; Q3 – upper quartile; SD – standard deviation; FU – follow-up. ***Notes:*** *repeated measures ANOVA.
